# Stride Lengths during Maximal Linear Sprint Acceleration Obtained with Foot-Mounted Inertial Measurement Units

**DOI:** 10.3390/s22010376

**Published:** 2022-01-04

**Authors:** Cornelis J. de Ruiter, Erik Wilmes, Pepijn S. van Ardenne, Niels Houtkamp, Reinder A. Prince, Maarten Wooldrik, Jaap H. van Dieën

**Affiliations:** Department of Human Movement Sciences, Faculty of Behavioural and Movement Sciences, Vrije Universiteit Amsterdam, 1081 BT Amsterdam, The Netherlands; e.wilmes@vu.nl (E.W.); Pepijnarden@gmail.com (P.S.v.A.); n.houtkamp@student.vu.nl (N.H.); r.a.prince@student.vu.nl (R.A.P.); m.wooldrik@student.vu.nl (M.W.); j.van.dieen@vu.nl (J.H.v.D.)

**Keywords:** wearables, IMU, stride length, running, acceleration, athletics

## Abstract

Inertial measurement units (IMUs) fixed to the lower limbs have been reported to provide accurate estimates of stride lengths (SLs) during walking. Due to technical challenges, validation of such estimates in running is generally limited to speeds (well) below 5 m·s^−1^. However, athletes sprinting at (sub)maximal effort already surpass 5 m·s^−1^ after a few strides. The present study aimed to develop and validate IMU-derived SLs during maximal linear overground sprints. Recreational athletes (*n* = 21) completed two sets of three 35 m sprints executed at 60, 80, and 100% of subjective effort, with an IMU on the instep of each shoe. Reference SLs from start to ~30 m were obtained with a series of video cameras. SLs from IMUs were obtained by double integration of horizontal acceleration with a zero-velocity update, corrected for acceleration artefacts at touch-down of the feet. Peak sprint speeds (mean ± SD) reached at the three levels of effort were 7.02 ± 0.80, 7.65 ± 0.77, and 8.42 ± 0.85 m·s^−1^, respectively. Biases (±Limits of Agreement) of SLs obtained from all participants during sprints at 60, 80, and 100% effort were 0.01% (±6.33%), −0.75% (±6.39%), and −2.51% (±8.54%), respectively. In conclusion, in recreational athletes wearing IMUs tightly fixed to their shoes, stride length can be estimated with reasonable accuracy during maximal linear sprint acceleration.

## 1. Introduction

Stride length can be estimated with inertial measurement units (IMUs) attached to the feet. IMUs are small sensors that measure triaxial accelerations and angular rates and often also include a magnetometer to determine orientation. Most research has focused on walking and reports (highly) accurate estimates of SL with only a few percent error compared to a golden standard [1,2,3,4].

In most sports, running is more important than walking. Together with stride frequency, SL determines acceleration and maximal speed, which are both important performance determining factors not only in athletics [5], but also in team sports like football [6]. Therefore, optimization of SL (and frequency) is of interest to athletes, coaches, and scientists and, consequently, it has been measured in many studies on performance [5,7,8,9,10,11,12] and training [13,14,15,16]. To this end, video or optoelectronic systems have commonly been used and, in general, these require an unobstructed view of the athlete, are relatively expensive, and analysis can be quite laborious. Obviously, direct determination of SL from IMUs attached to the feet of athletes, similar to what has been done during walking, would be of great practical benefit for training and testing in sports.

SL determination from sensor signals during running may be far more challenging than during walking. During running, the participants and, consequently, any sensors attached to them, move much more vigorously, which introduces large accelerations and fast changes in sensor orientation [17]. In particular, the high impacts upon touch-down of the feet may introduce an additional challenge for the double integration procedures of acceleration that are commonly used to derive SL. Consequently, only a limited number of studies determined SL with IMUs and did so during running at constant and relatively low speeds (e.g., [17,18,19,20]). Zrenner et al. found that errors in estimated SL increased greatly from 5 to 6 m·s^−1^_,_ which, to the best of our knowledge, is the highest speed interval for which IMU-derived SLs have been reported [17].

During maximal linear sprint acceleration, there are several factors that will complicate SL determination compared to running at constant submaximal speeds. There will be (participant-dependent) changes in running technique during the transition from the acceleration phase to full speed sprinting [7,8,21,22]. Moreover, compared to running at a constant speed, greater variations in foot placement are expected. Touch-down may, for example, occur slightly outside the sagittal plane during the first strides. Most importantly, the upper limit (5 m·s^−1^) of investigated speeds for which SL has been accurately derived from IMUs is rather low. During sprint acceleration, 5 m·s^−1^ is reached after the first few strides [10,23] and recreational athletes reach maximal speeds of about 9 m·s^−1^ [10].

Recently, we demonstrated that fairly accurate estimates of SL could be obtained during maximal sprint acceleration [10], but these estimates were indirectly obtained from the IMU-signals by combining the timing of the footfalls with the well-known mono-exponential speed increase during sprint acceleration [23]. To the best of our knowledge, there are no publications in which SL is determined directly from the IMU signals during maximal linear sprint acceleration.

In walking and running, zero velocity updates are a common procedure when SL is determined from double integration of the acceleration signals [1,2,17,19]. Zero velocity updates are made under the assumption that the sensor stands still at some point during ground contact. It has been argued that this assumption may not hold during running at high speed [17]. Nevertheless, even in maximal sprinting there is a short time window in the early stance phase (during amortisation) immediately following touch-down, where sensor velocity will be (close to) zero. Moreover, in pilot measurements with a few athletes and using the assumption that sensor velocity was zero at 20% of ground contact time, we obtained SLs that appeared to be fairly accurate. This was our incentive for a more systematic investigation.

The objective of the present study was to develop an algorithm that accurately estimates SLs during (maximal) acceleration across a range of running speeds that are much faster than those investigated thus far. We hypothesised that accurate determination of SL during sprints executed at different percentages of maximal effort (60–100%) is possible when touch-down-related artefacts in the IMU signals are corrected for.

## 2. Materials and Methods

### 2.1. Participants

The study was conducted according to the guidelines of the Declaration of Helsinki, and approved by the ethics committee of the Faculty of Behavioural and Movement Sciences of the Vrije Universiteit (VCWE 2016181R1). After signing informed consent and after being informed about the goals and procedures, twenty-one recreational track and field athletes from two different local clubs participated (Table 1). They were free from injury and were individually tested on the outdoor tartan running track at their club. Eighteen athletes wore spiked shoes and three of the male participants wore running shoes.

### 2.2. Test Procedures

The participants did their own pre-competition warm-up, which included acceleration runs to maximal speed. Subsequently, a custom built (35 × 25 × 10 mm, 11 g) 9-DOF IMU (MPU−9150, ±16.0 g, ±2000°/s, ±1200 μT, Invensense, San Jose, CA, USA) was attached to each shoe. Prior to that, IMUs were switched on and tapped together on a hard surface for time-synchronization [10,24]. The IMUs continued to sample for the entire experimental session and the data were logged (500 Hz sample frequency) on an internal SD-card for off-line analysis, as described in detail elsewhere [10,24].

**Figure 1:**
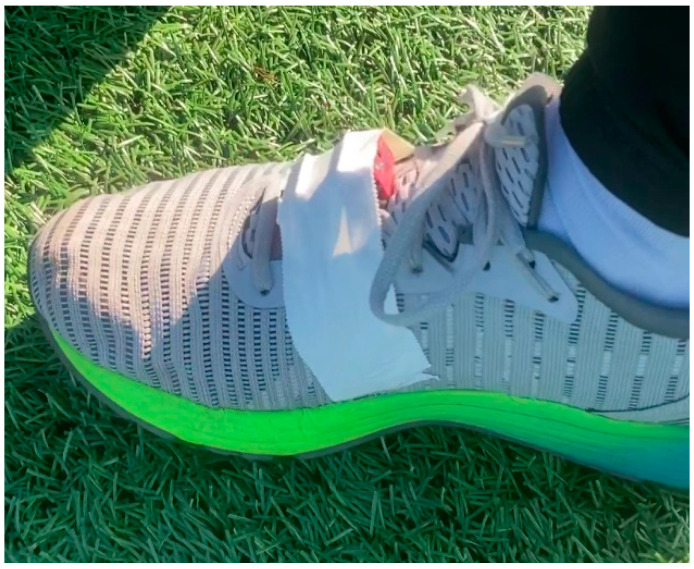
Attachment of the IMU on the instep.

IMUs were secured on the instep of each shoe using a tight elastic band placed under the shoelaces and hooked over the clips of the IMU (Figure 1). The IMUs were further secured with an additional cross strap of sports tape. A stride was defined from touch-down of one foot to the subsequent touch-down of the same foot. The strides of both feet were analysed. Participants started from a split stance position with their front foot placed against the start line. The strides of this foot were denoted ‘front foot’ strides and that of the other, ’rear foot’ strides.

Following warm-up and IMU-attachment, the participants ran two sets of three sprints starting every 3 min. In each set, the sprints were done in order of increasing intensity and executed at about 60%, 80%, and 100% of maximal effort.

The participants started at their own initiative. They ran on a straight white line of the track in between two parallel lines (1 m distance in between) of small cones. The cones in each line were placed at every 3 m and used for video calibration. The touchdowns of the runners’ feet were video recorded (30 Hz) from the side with 5–6 cameras (Fujifilm XP60 Full-HD) mounted on tripods (1.2 m height) and placed 8 m to the side of the sprint lane, from 3 to 27–33 m at 6 m intervals. To determine SL from video, the tips of the shoes were manually digitized for all recorded steps [10] using free software (Kinovea 0.8.15 www.kinovea.org; (accessed on 8 June 2021). Stride lengths were divided by IMU-derived stride durations (see below) to obtain stride speeds [10]. The highest stride speed reached was defined as the maximal stride speed.

### 2.3. Analysis of the IMU Data

The instances of touch-down and toe-off of both feet were derived from the IMU-signals. Specifically, the gyroscope of the sensors’ x-axis (medial–lateral foot direction) was used for automized detection of the exact instances of touch-down and toe-off, as described in detail elsewhere [10]. Subsequently, IMU orientation relative to the global reference frame was determined using a gradient descent Madgwick algorithm with the gain (beta) set to 0.043 [25]. The global reference frame was defined with the y-axis pointing upward, the x-axis pointing in the direction of the horizontal component of the earth magnetic field vector, and a transverse z-axis [24].

The participants started from standstill and, hence, the first toe-off of each foot lacked a preceding touch-down event; therefore, for both feet, we had to decide how many samples before the first toe-off we would start the double integration procedure to derive length of the first stride. We established that using a 280 ms interval before toe-off included all visible (in the signals) movements of both feet before toe-off for all participants, regardless the differences in split stance position and start execution among the athletes.

**Figure 2:**
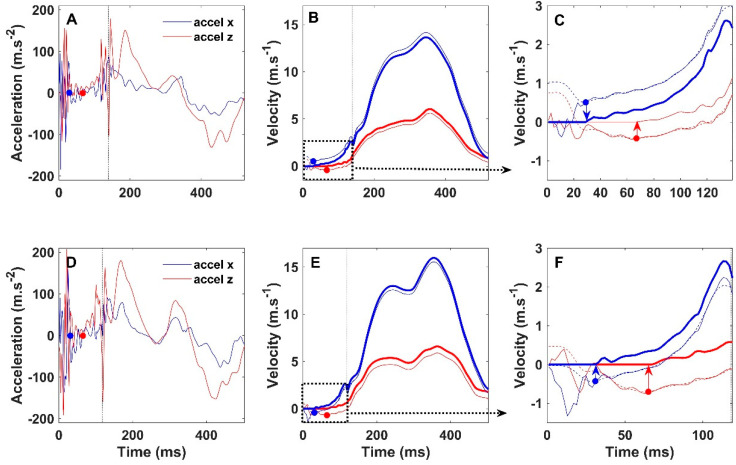
Acceleration (**A**,**D**) and velocity signals in the horizontal plane (x-z) of two strides within the same sprint (participant 2, right foot) are depicted. Top panels show stride number four: stride speed 7.2 m·s^−1^, SL 3.70 m from video, and 3.72 m from IMU. Bottom panels show stride number nine: stride speed 8.8 m·s^−1^, SL 4.41 m from video, and 4.36 m from IMU. Touch-down occurs at 0 s and the vertical dotted lines near 130 ms indicate the moments of toe-off. First, the unfiltered acceleration signals of a complete stride (**A**,**D**) were integrated and mirrored in cases where average stride velocity was negative. This resulted in the ‘original’ velocities (thin traces in (**B**–**F**)) of which sample nos. 1 to 10 were given the value of the tenth sample (20 ms), to which 1 m·s^−1^ was added. This procedure guaranteed that the velocity signals would always decline after 20 ms. Together with the 40 Hz low-pass filtering, this resulted in ‘temporary’ (dashed traces) velocity signals in which a minimal value could be found in a consistent manner. These minimal values were used for offset determination. The offsets are indicated by the dots and the arrows in (**C**,**F**), which, respectively, are enlargements of the first part of the traces in (**B**,**E**). The velocity offsets were subtracted from the ‘original’ velocity signals and data points before the moment of offset detection were given the value zero. This resulted in the ‘final’ offset-corrected velocity signals (thick traces in (**B**–**F**)) in the x (red) and z (blue) direction. The latter were integrated to determine displacements in the x and z direction, the norm of the resultant vector being stride length.

All subsequent toe-off events were preceded by a touch-down that caused large peaks in the acceleration signals, which, upon integration, led to an offset in velocities in the horizontal (x, z) plane during the first half of the ground contact phase (Figure 2). These velocity offsets were considered to be caused by acceleration artefacts at touch-down and had to be corrected (zero velocity updates) before performing a second integration to obtain displacement vectors in the x and z direction. This correction procedure is explained in detail in the caption of Figure 2. The velocity offsets were determined by searching the minimal value in the filtered (‘temporary’) velocity signals between 20 and 100 ms following touch-down. For zero velocity updating, we aimed to obtain the first index after the touch-down artefacts that would be followed by a consistent increase in the horizontal (x, z) velocities. To remove the effects of high frequency oscillations on this procedure, the velocity signals were 40 Hz low-pass filtered. These high frequency oscillations were assumed to be related to sensor vibrations caused by the landing impact. For all second strides, during which sprint speed is still low and which have the longest ground contact time of all strides (usually >150 ms), the standard window for offset detection (20–100 ms) was set to 20–160 ms after touch-down. This longer window was found to reduce stride error estimation for the second stride of both feet in many participants.

In the examples of Figure 2, the velocity offsets are indicated by two dots with additional arrows, in the panels on the right. The minimal ‘temporary’ velocity values indicated by the dots were used for zero velocity updating, which led to an up or downward shift of the velocity signals as described in the legend of Figure 2 and as indicated by the direction of the arrows (Figure 2C,F).

This procedure resulted in (unfiltered) offset-corrected velocity signals (respectively red and blue for x and z direction in Figure 2) that were integrated over the complete stride to obtain displacements in the x and z direction. The norm of these two displacement vectors was calculated to obtain stride length. As speed increases, the landing impacts change and, consequently, even within participants, the velocity offsets change over strides and between sprints. This is also shown in Figure 2, where the offset in z-velocity is positive for stride 4 (blue arrow down in C), while it is negative in stride 9 (blue arrow up in F).

### 2.4. Statistical Analysis

Statistical analysis was done using Matlab (version 2018b, The MathWorks, Inc., Natick, MA, USA) and R [26]. Normal distribution of the data was confirmed by visual inspection of the histograms, Q–Q plots, and with the Shapiro–Wilk test. To investigate whether sprint effort (speed) and stride number (stride number one is the first stride following the start) affected validation, linear mixed effect regressions (lmers) were performed with effort (60–80–100%), stride number (1–9), and method (video vs. IMU) as fixed factors and with participants as a random factor to account for the differences between subjects. Effect sizes are reported as partial eta squared values (ƞ^2^). The similarity between SLs obtained from video and IMUs was also investigated with Bland–Altman (BA) analyses (bias ± limits of agreement (LOA)). The BA procedure that was used accounted for the repeated measures within participants [27].

## 3. Results

Maximal stride speeds (mean ± SD) reached near the 30 m mark during sprints executed at 60, 80, and 100% effort, respectively, were: 7.02 ± 0.80 m·s^−1^, 7.65 ± 0.77 m·s^−1^, and 8.42 ± 0.85 m·s^−1^ (*p* < 0.05). The respective mean values of the reference SLs obtained from video were: 3.49 ± 0.76 m, 3.46 ± 0.79 m, and 3.33 ± 0.75 m. Stride length depended on all investigated factors. There were significant main effects on SL of execution effort (*p* < 0.001, ƞ^2^ = 0.16), method (video or IMU; *p* < 0.001, ƞ^2^ = 0.009), and stride number (*p* < 0.001, ƞ^2^ = 0.91). In addition, the following significant interaction effects were present: execution level x method (*p* < 0.001, ƞ^2^ = 0.007), execution level x stride number (*p* < 0.001, ƞ^2^ = 0.04), and method x stride number (*p* = 0.02, ƞ^2^ = 0.004).

### 3.1. Effects of Sprint Effort on SL Estimation

**Figure 3:**
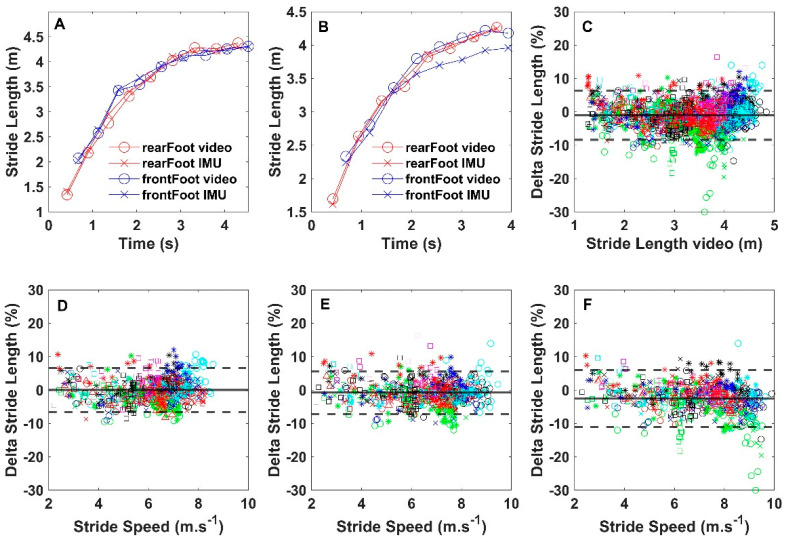
Typical examples of SL of both feet (rear foot at the start in red, front foot in blue) as a function of time for participant nos. 3 (**A**) and no. 9 (**B**) at 100% effort reaching maximal speeds of 9.17 and 8.95 m·s^−1^, respectively. Note that SLs from video (open circles) and IMUs (crosses) are very similar for both feet in (**A**), while in the other participant, the IMUs underestimated SL compared to the reference for the front foot only (**B**). (**C**) depicts the delta values (IMU–video) expressed as a percentage of video SL for all strides as a function of the video reference. Each symbol represents the data of a single participant. Delta values from sprints executed at 60, 80, and 100% effort, respectively, are shown (**D**–**F**) as a function of stride speed. Note that already at 60% effort, most strides were executed at a speed >5 m·s^−1^. The continuous lines represent the biases and the dashed lines, the limits of agreement.

Bland–Altman analysis (Figure 3C) reveals a small negative bias of −1.07% (LOA 7.33%) for IMU-derived SL compared to the video reference when all 2226 strides are included. Percentage error is plotted as a function of stride speed for each level of effort separately in Figure 3D–F. The biases (±LOA) for SLs obtained during sprints executed at 60, 80, and 100% effort, respectively, were 0.01% (±6.63%), −0.75% (±6.39%), and −2.51% (±8.54%). IMU-derived SLs were not different from video-derived SLs at 60% effort (*p* = 0.75). However, at 80% (*p* = 0.006) and 100% effort (*p* < 0.001), IMU-derived SLs differed significantly from video-derived SLs, but the respective effect sizes were small (ƞ^2^ = 0.005 and 0.05).

### 3.2. Effects of Stride Number on SL Estimation

Within sprints, the SLs increased significantly with stride number (*p* < 0.001), with large effects on sizes at all three levels of execution effort (ƞ^2^ > 0.91). This illustrates the well-known increase in SL during sprint acceleration. At 60% and 80% effort, the interaction effect between stride number and method (video or IMU) was not significant (*p* > 0.77). However, at 100% effort, this interaction was significant (*p* = 0.0005) and, although the effect size was small (ƞ^2^ = 0.02), this signifies that within sprints executed at 100% effort, the error of IMU-derived SL increased with stride number. There were four participants in particular (the green symbols in Figure 3) for whom SLs were underestimated with the IMUs. This underestimation generally became more pronounced when the sprints were executed at 100%. At 100% effort, relative stride error (% video reference) was found to increase during the later strides of the sprints. This can be seen in the BA plots of the separate effort levels in Figure 3D–F, where the relative differences between methods are plotted as functions of stride speed and most of the negative outliers reside in Figure 3F. For several participants, the magnitude of the underestimation of SL during the later strides clearly differed between feet; for several others, the underestimation was only found in one of the feet (e.g., Figure 3B). The data points on the left side of the bottom panels in Figure 3 are from the first strides during the sprints and, because speed increases exponentially during acceleration, the majority of SLs were obtained at speeds above 5 m·s^−1^ at each of the three levels of effort.

## 4. Discussion

The results of the present study show that it is possible to derive reasonably accurate estimates of SL during maximal sprint acceleration using foot-mounted IMUs. The errors in estimated SLs of the three female sprinters were within the range of the male sprinters. The accuracy is comparable, with the results reported in the literature for runs executed at constant and much slower speeds. Brahms et al. found good agreement (bias −3.2 cm and LOA of 15 cm) between SLs estimated with a foot-mounted IMU and a gold standard motion capture reference during runs at constant speed in a laboratory setting [19]. For sprints executed at 60% effort in the present study, the bias was smaller (−0.46 cm), but the LOA were wider (23 cm). The set-up of the present study has a high ecological validity, but, as a consequence, we had to use video as a reference. Reference SLs obtained from video are subject to error (imperfect camera angles, digitization errors). The manual digitization of videos introduced a random error of a few cm in our reference step lengths. If we could have used a true gold standard reference, the limits of agreement might have been smaller, but this speculative. More importantly, average running speed in the study of Brahms et al. only was 3.55 m·s^−1^ (range 2.72–4.36 m·s^−1^) and SLs were considerably smaller, and their LOA as a percentage of average SL (0.15 m/2.59 m = 5.8%) were comparable to the 6.6% found in the present study. Moreover, in the present study, average peak speed near the 30 m mark was much higher (7.02 ± 0.80 m·s^−1^) and 649 of the 735 strides during sprints executed at 60% effort had stride speeds beyond the range investigated by Brahms et al. Together with the inclusion of the acceleration phase, this increased the challenge for accurate SL estimation in the present study. Zrenner et al. also didn’t include the acceleration phase in their runs, but they investigated SL estimations from foot-mounted IMUs over a wider speed range (2–6 m·s^−1^) compared to Brahms et al. [17]. During sprints executed at the lowest effort (60%) in the present study, still, 453 of the 735 analysed strides were obtained at speeds faster than the upper limit of 6 m·s^−1^ of Zrenner et al. Nevertheless, our bias (−0.46 cm) and LOA (0.23 m) are very similar to theirs (their Figure 8e [17]). They also reported that accuracy of their estimations dropped considerably between 5 and 6 m·s^−1^ [17], which underlines the importance of accounting for speed in the comparisons between studies. Note that, with further increases of speed during sprints executed at 80% effort, the increase in estimated SL error was limited. So, clearly, the algorithm proposed here expands the reported speed range for which SL can be accurately estimated with IMU sensors mounted on the shoes. However, for some participants, relatively large errors were found in the present study, particularly when sprinting at 100% effort. There are several potential causes for these errors that need to be addressed.

Participant number 1 (open green circles in Figure 3) was the only participant for whom stride length was systematically underestimated in both feet and at all three levels of sprint effort. The foot angle time profiles for this participant (not presented) clearly showed deviations that increased with sprint speed (effort), and these deviations were larger in the foot displaying the greatest error. This finding of imperfect sensor orientation estimations (foot angle) may have been caused by suboptimal sensor fixation. In addition, the videos indicated that the sensors were placed rather distally on the shoe and perhaps too close to the toes. Shoe deformation just prior to toe-off may have caused additional accelerations of the sensor, leading to inaccurate SL estimates following integration. In the present study, the participants ran in their own shoes. The shoes being of different brands/types complicated standardization of sensor fixation on the instep. Some shoes, for example, had an additional cover with a zipper over the shoelaces and/or an additional strap over the instep.

Besides participant number 1, there were seven other participants for whom the derived foot angle–time profiles of at least one of the feet showed indications of suboptimal sensor fixation. In these cases, the underestimation of SL generally increased during the later strides of sprints executed at 100% effort, which accounts for the small but significant negative bias of −2.51% for sprints executed at 100% effort. At maximal effort, average peak speed and stride frequency were respectively 10% and 15% higher compared to sprints executed at 80% effort. The more vigorous movements while sprinting at 100% effort may have increased SL estimation error in these seven participants. However, sprint speeds and stride frequencies of these seven participants were similar to those attained by other participants sprinting at 100% effort. Moreover, in several participants, the error increased in only one foot (e.g., Figure 3B). Taken together, these findings suggest that, despite our effort to tightly fixate the sensors, the fixation may still have been suboptimal in some cases. The importance of sensor fixation was recently demonstrated by Zrenner et al., who found the lowest errors with IMUs integrated into the sole of a standard type of shoe [20]. Although we do believe that sensor fixation has affected our results, the extent to which this was the case remains unclear. Moreover, we cannot exclude that the proposed algorithm simply did not work well on some data sets. Therefore, all strides of all participants were analysed, including the sets where sensor fixation, in all likelihood, must have been suboptimal. For future research, we advise avoiding fixation of the sensors too close to where the shoes might deform prior to toe-off.

In addition to the problems with sensor fixation, the velocity correction procedure that was used may have introduced some errors. We assumed that the large peaks in the acceleration signals in the first 20 ms of ground contact do not signify meaningful accelerations of the foot in the horizontal plane. However, if ‘real’ horizontal acceleration of the feet does occur within this 20 ms time window, our velocity correction will be suboptimal. This may have contributed to the generally small underestimation of SL when some of our participants sprinted at maximal effort. When sprint speed approaches maximal values, stride frequency increases and ground contact time decreases, e.g., [10]. Therefore, it also remains to be seen if SL can be determined with the current algorithm in faster athletes. Due to the limited measurement ranges of our current sensors, we could not test the stride length estimation algorithm on the data of more proficient sprinters. In several instances, the angular rates approached the sensors’ limits in the present participants and acceleration limits already were often surpassed during the first 20 ms of ground contact. Obviously, the latter contributed to the necessity to ignore the first 20 ms of ground contact in the calculations of SL.

Ignoring the sensors’ accelerations during the first 20 ms of ground contact also prohibited additional dedrifting prior to the integration of velocity to displacement. The acceleration (and angular rate) signals are subject to inherent noise leading to errors that amplify during integration, causing errors in estimated velocity and, consequently, SL. To correct for integration drift, usually some kind of (linear) dedrifting technique is used that assumes that deviations of velocity from zero following integration of acceleration over one complete stride must be due to integration drift and should be corrected for [17]. Ignoring the first 20 ms invalidated using commonly used dedrifting methods. The velocity at touch-down also could not be used to correct for integration drift because the exact velocity of the foot at that instant is unknown. Of note, when we included the first 20 ms of acceleration after touch-down to estimate SL, large errors in SL estimation occurred that did not decrease following linear dedrifting. Thus, although integration drift certainly contributed to errors in the estimated length of individual strides, the effect does not seem to be large.

Despite the critical notes made in the foregoing paragraphs, it is important to note that the errors in estimated SL are comparable to those reported in the literature for (much) slower runs executed at constant speed. We believe that the accuracy of estimated SL is good enough to be of practical relevance. Moreover, measurements with IMUs can easily be done without interfering with the athletes’ training session and the analysis is completed within a few minutes. In addition, many measurements can be made per individual athlete, which may further reduce random error and will provide athletes and coaches with the changes in SL and stride frequency (and, therefore, running speed) in response to training or during rehabilitation from injury. The use of wireless transmission would even make it possible to provide immediate feedback during training sessions.

We conclude that reasonably accurate estimation of SL with foot-mounted IMUs during maximal linear sprinting is possible in recreational athletes. However, it remains to be established if, with the use of sensors with larger bandwidths, further improvement of sensor fixation and/or adaptation of the algorithm will suffice for accurate estimations of SL in more proficient sprinters.

## Figures and Tables

**Table 1:** Participants’ characteristics.

ID No.	Age (Years)	Mass (kg)	Height (m)	100 m Time (s)	Sex (m/f)	Shoe Type
1	16	71	1.82	12.18	m	spiked
2	19	78	1.87	11.56	m	spiked
3	18	79	1.91	11.40	m	spiked
4	18	68	1.85	11.92	m	spiked
5	19	71	1.80	11.69	m	spiked
6	17	70	1.85	11.95	m	spiked
7	23	89	1.88	11.33	m	spiked
8	19	65	1.87	13.10	m	spiked
9	20	80	1.93	11.91	m	spiked
10	31	62	1.75	11.57	m	spiked
11	27	70	1.69	12.42	v	spiked
12	22	60	1.67	13.48	v	spiked
13	29	76	1.88	11.52	m	spiked
14	36	72	1.92	12.47	m	spiked
15	20	67	1.83	12.61	m	spiked
16	24	72	1.66	15.90	v	spiked
17	21	73	1.79	12.41	m	spiked
18	26	72	1.88	12.97	m	spiked
19	36	65	1.68	14.10	m	running
20	22	85	1.85	13.17	m	running
21	22	72	1.71	13.08	m	running
mean	23.1	72.2	1.81	12.51		
SD	5.8	7.1	0.09	1.08		

## Data Availability

Doi: 10.5281/zenodo.5814971. Description: Individual raw data files of the separate sprints, including the instances of toe-off and touch-down and the acceleration data in the global references frame.
